# PROC QTL—A SAS Procedure for Mapping Quantitative Trait Loci

**DOI:** 10.1155/2009/141234

**Published:** 2009-12-08

**Authors:** Zhiqiu Hu, Shizhong Xu

**Affiliations:** Department of Botany and Plant Sciences, University of California, Riverside, CA 92521, USA

## Abstract

Statistical analysis system (SAS) is the most comprehensive statistical analysis software package in the world. It offers data analysis for almost all experiments under various statistical models. Each analysis is performed using a particular subroutine, called a procedure (PROC). For example, PROC ANOVA performs analysis of variances. PROC QTL is a user-defined SAS procedure for mapping quantitative trait loci (QTL). It allows users to perform QTL mapping for continuous and discrete traits within the SAS platform. Users of PROC QTL are able to take advantage of all existing features offered by the general SAS software, for example, data management and graphical treatment. The current version of PROC QTL can perform QTL mapping for all line crossing experiments using maximum likelihood (ML), least square (LS), iteratively reweighted least square (IRLS), Fisher scoring (FISHER), Bayesian (BAYES), and empirical Bayes (EBAYES) methods.

## 1. Introduction

The discovery of genes that contribute to the expression of complex traits is one of the fundamental and essential tasks in genetic research. In the past decades, many QTL mapping procedures have been developed. A larger number of computer programs are now available to implement these methods. These programs have significantly simplified the applications of the methods to the downstream genetic research. A complete list of the programs is posted on the web sites http://linkage.rockefeller.edu/soft and http://www.stat.wisc.edu/~yandell/statgen/software/biosci/linkage.html. The software package presented here (Version 1.0) is available at http://www.statgen.ucr.edu/software.html.

Most of the programs were developed as standalone software packages. These include MapMaker/QTL [1], Map Manager [2], QTL Express [3], MapQTL [4], MCQTL [5], MULTIMAPPER [6], MetaQTL [7], WinQTLCart [8], and QTLNetwork [9]. Other programs were developed using the R package, for example, R/qtl [10] and R/qtlbim [11]. PROC BTL is a trial version of a SAS procedure for mapping binary trait loci (BTL) [12]. Each of the aforementioned programs was developed targeting on one or a few jobs. In addition, these programs were designed by different programmers, and they usually require users to provide data in very restricted formats. Users probably need to prepare their data using different formats before they can switch among the different programs, especially when both continuous and categorical traits are involved. We now report a new software package called “PROC QTL” that was recently developed by the Quantitative Genetics Group at UC Riverside. This software package can perform QTL mapping in almost all line crossing experiments for both quantitative and categorical traits.

## 2. Features

PROC QTL was coded in C++ and the interface with the SAS system was conducted using the SAS/Toolkit software [12]. PROC QTL is different from other stand alone QTL mapping software packages, in that the program must be executed within the SAS system to perform all the QTL analysis. Once PROC QTL is installed, users can call the procedure just like they call any other regular SAS procedures without noticing the differences between this customized procedure and other built-in SAS procedures. The SAS system provides services to the procedure such as statement processing, dataset management, and memory allocation. PROC QTL can read SAS datasets and data views, perform data analysis, print results, and create other SAS datasets. 

There are many advantages to perform QTL mapping under SAS rather than using stand-alone programs. A few are listed here. (1) Familiarity: using PROC QTL is easy for SAS users because they already understood the general SAS syntax, data input, data output, and other kinds of data manipulation. (2) Integration: the data used by PROC QTL can easily be sorted, printed, analyzed and plotted using other SAS procedures during a single job. (3) Special capabilities: special features, such as BY-group processing and Weight variable handling, can be used in PROC QTL. (4) Reduced documentation: only the new language statements, the output of the procedure, and any special calculations in the procedure need to be explained.

## 3. Methods and Algorithms

With PROC QTL, users can perform QTL mapping for both continuous traits and categorical traits in line crosses, including F_2_, BC (backcross), RIL (recombinant inbred lines), DH (doubled haploid), and FW (four way crosses). For continuously distributed traits, there are six methods that users can choose from. These methods include maximum likelihood [13], least squares [14], iteratively reweighted least square [15, 16], Fisher scoring method [17], Bayesian method [18, 19], and an empirical Bayesian method [20]. For categorical traits, the generalized linear model (GLM) is used as the basic framework of data analysis. Due to the problem of missing genotypes in QTL mapping, the GLM is implemented in several different ways, including the expectation substitution method, the heterogeneous residual variance method, and the mixture distribution method (the EM algorithm). PROC QTL can also handle epistatic effects (allelic interactions between loci) under the empirical Bayesian method.

## 4. Implementation

PROC QTL is a SAS procedure and thus can be called within the SAS environment. It takes SAS datasets as input data and output results as SAS datasets. Therefore, data preparation and manipulation of PROC QTL are conducted using SAS data steps or other built-in SAS procedures. 

**Figure 1:**
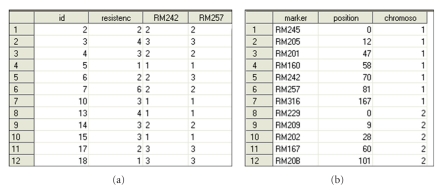
Dataset structure for the ricedata (a) and ricemap (b).

The following gives the SAS code to call PROC QTL for mapping a disease resistance trait using the data provided by Zou et al. [21]. The resistance of rice to sheath blight disease was measured in grades ranging from 1 to 6 for 119 rice plants sampled from an F2 population. There are 12 molecular markers, distributed along two chromosomes covering 268 cM in length. By using data manipulating modules of SAS software such as DATA STEP or DATA WIZARD, users can easily import/export their data from/to Microsoft excel, text file, Lotus spreadsheet, and various other commonly used file formats. The dataset prepared for this example is shown in Figure 1.

We can perform QTL mapping using the EM implemented maximum likelihood method. The code is demonstrated as follows:

**proc qtl** data= ricedata map= ricemap out=result
method=“ML”;
 class resistenc;
 model resistenc = ;
 matingtype “F2”;
 genotype A=“1” B=“3” H=“2”;
 estimate “a”=1 0 -1;
 
**run**:

The result produced by RPOC QTL will be saved in the SAS dataset specified in the “out=” option. User can conveniently export or plot the result using other standard SAS procedure, for example, PROC GPLOT. The following SAS code shows how to generate LRT profile using the result dataset produced by PROC QTL:

symbol1 interpol=join value=none;
**proc gplot** data=result;
 plot lrt*position;
 by chr;
**run**;

Four more examples are automatically made available once PROC QTL is installed. The SAS code and datasets for the additional examples will be automatically copied to users' computers.

## 5. Future Development

PROC QTL is a platform on which more options can be added. The current version of PROC QTL can only be run in Windows. The Unix version is under development and will be released soon.
